# New hematologic populations at risk of invasive aspergillosis: focus on new targeted, biological, and cellular therapies

**DOI:** 10.12688/f1000research.17836.1

**Published:** 2019-07-26

**Authors:** Corrado Girmenia

**Affiliations:** 1 Dipartimento di Ematologia, Oncologia, e Dermatologia, Azienda Policlinico Umberto I, Sapienza University of Rome, Rome, Italy

**Keywords:** invasive aspergillosis, hematologic malignancies, targeted treatments

## Abstract

The introduction of new targeted, biological, and cellular therapies in patients with hematologic malignancies has improved the outcomes of patients but in parallel has changed the frequency and epidemiology of infections, including invasive aspergillosis (IA). In this article, recent literature on the epidemiology and clinical findings of IA in patients who have lymphoproliferative and myeloproliferative diseases and are undergoing novel targeted treatment with kinase inhibitors, agents targeting cell surface antigens, chimeric antigen receptor-modified T cells, and antibodies to immune checkpoint molecules is reviewed and the clinical impact of IA on the overall management of the underlying disease is discussed. Overall, IA represents a variable and uncommon complication in these populations, but given the increasing eligibility criteria of these novel treatments (particularly in patients with relapsed or refractory hematologic malignancies) and the prolonged periods of therapy, a considerable number of unusual cases of *Aspergillus* infections can be expected in clinical practice.

## Introduction

Invasive aspergillosis (IA) occurs infrequently among immunocompetent individuals but is a major infectious complication in immunocompromised patients, particularly those affected by hematologic malignancies, because of its high incidence and associated mortality ^1– 3^. Historically, acute leukemia (AL) and allogeneic hematopoietic stem cell transplant (allo-HSCT) represent the underlying conditions that put patientsat higher risk of IA, and most epidemiological studies and clinical trials of prophylaxis and therapy of IA involved these hematologic populations ^4– 6^. However, in recent decades, several studies have shown a change in the epidemiological patterns of IA with an increasing involvement of patients with other hematologic malignancies (OHMs) (that is, other than AL and allo-HSCT). Of 109 infections in a retrospective study of consecutive episodes of proven/probable invasive pulmonary aspergillosis diagnosed at an Italian hematologic center from 2006 to 2011, 51% were diagnosed in patients with AL, 20% in patients who underwent allo-HSCT, and 28% in those affected by lymphoproliferative diseases ^7^. Several national registries confirmed that OHMs represent frequent underlying conditions in patients with diagnosed IA ^8– 10^. According to the results of the Global Burden of Disease Study on cancer populations in 192 countries and territories, for each case of AL (acute myeloid leukemia and acute lymphoid leukemia), 5.3 cases of OHM (lymphoma, multiple myeloma, and chronic leukemias) were diagnosed in 2016 ^11^. Nevertheless, although the overall incidence of IA in patients with OHM is less than 2% ^12, 13^, the number of cases of IA encountered in clinical practice in patients with OHM is highly relevant given the high number and prolonged survival of these patient populations.

Recent data show a different spectrum of infectious complications in patients with lymphoid and myeloid diseases treated with new targeted, biological, and cellular therapies with documented cases of invasive fungal diseases (IFDs) in certain populations ^14– 19^. However, most initial studies evaluating the efficacy and safety of novel treatments are performed in patients with a relapsed or refractory disease, making it difficult to establish the additional risk of infections conferred by novel agents and the effect of previous treatments, concurrent complex immunosuppression, and the role of the underlying disease.

In this article, recent literature on the epidemiology and clinical findings of IA in patients undergoing new targeted, biological, and cellular therapies is reviewed and the clinical impact of this complication on the overall management of the underlying disease is discussed.

## Bruton’s tyrosine kinase and phosphatidylinositol-3-kinase inhibitors

Advances in the understanding of B-cell receptor (BCR) signaling and its role in promoting B-cell survival and proliferation have highlighted new targets for the treatment of chronic lymphocytic leukemia (CLL) and certain non-Hodgkin lymphomas (NHLs). Ibrutinib is a first-in-class Bruton’s tyrosine kinase (BTK) inhibitor that is indicated for use in adult patients with CLL, Waldenström’s macroglobulinemia, mantle cell lymphoma, and marginal zone lymphoma as single-agent therapy or in combination with other drugs ( https://www.ema.europa.eu/documents/product-information/imbruvica-epar-product-information_en.pdf). Idelalisib is a first-in-class inhibitor of phosphatidylinositol-3-kinase delta (PIK3-delta), another component of the BCR signaling pathway, and is indicated in combination with an anti-CD20 monoclonal antibody (rituximab or ofatumumab) for the treatment of CLL in adult patients and as monotherapy for the treatment of follicular lymphoma in adult patients ( https://www.ema.europa.eu/documents/product-information/zydelig-epar-product-information_en.pdf).

Both ibrutinib and idelalisib affect critical components of the immune system, as proven by the increased infection burden reported in patients with hereditary mutations of both BTK and PIK3-delta ^20, 21^. A key role for BTK in macrophage immune responses was demonstrated during experimental pulmonary aspergillosis indicating that ibrutinib blocks inflammatory responses to *A. fumigatus* in human macrophages through a BTK-dependent pathway ^22, 23^. For these reasons, the risk of infection in patients with CLL and NHL treated with these new kinase inhibitors is higher than the overall risk reported in CLL and NHL populations ^24, 25^.

Evidence supporting the potential for infectious complications by the agents affecting the BCR has been provided mostly by clinical trials where infections (frequently of the respiratory tract) occurred typically at the beginning of treatment and the infection rate declined by more than half after a few months of therapy. However, specific epidemiological studies on infections treated with ibrutinib and idelalisib are scarce and most of the data derived from retrospective studies or from phase II or III clinical trials performed to test the efficacy and overall safety of these therapeutic agents but without detailed information on the infectious complications. An association with pulmonary IA was observed shortly after ibrutinib was licensed for use. Of 127 patients with relapsed or refractory CLL treated with ibrutinib with or without rituximab in a single-center study published in 2015, 33 (26%) discontinued treatment for different reasons. Overall, 14 of them discontinued ibrutinib because of adverse events or death, and in two cases a pulmonary IA was diagnosed ^26^.

Some real-life clinical series of patients who received ibrutinib as first-line or salvage therapy have recently been published along with detailed data on infectious complications. The spectrum of serious infections in 378 patients whose lymphoid malignancies were treated with ibrutinib from 2012 to 2016 at the Memorial Sloan Kettering Cancer Center was retrospectively reviewed ^27^. Overall, serious infection developed in 43 patients (11.4%), primarily during the first year of ibrutinib treatment, and IFDs were documented in 16 (37.2%). IFDs included proved or probable IA in eight patients and a concurrent probable IA and *Pneumocystis jirovecii* pneumonia in one patient. No patient was receiving an anti-fungal prophylactic regimen at the time of IA. The presence of neutropenia at any time during ibrutinib treatment and receipt of three or more previous anti-tumor regimens were significantly associated with an increased risk of severe infection. Specifically, the risk factor of corticosteroid use at any point during ibrutinib treatment was associated with the occurrence of IA. A single-institution retrospective study was carried out to find the type and incidence of opportunistic infections (OIs) during ibrutinib treatment and the characteristics and outcomes associated with risk ^28^. In 566 patients who received ibrutinib from June 2010 to March 2016 (74% of patients affected by CLL), the cumulative incidence of OI was 2.3% at 0.5 years and increased to 4.7% at 5 years. IFDs (mainly IA) accounted for 74% of OIs. In a multivariable analysis of at least three prior treatments (hazard ratio [HR] 2.87, 95% confidence interval [CI] 1.12–7.35; *P* = 0.028), diabetes (HR 3.63, 95% CI 1.50–8.77; *P* = 0.004) and liver disease (HR 7.53, 95% CI 2.14–26.49; *P* = 0.002) retained an independent association with OI development.

A multicenter survey aimed at identifying cases of IFD in patients whose CLL was treated with ibrutinib was conducted in France ^29^. Out of 33 IFDs, 27 were proven, probable, or possible IA with cerebral localization in 40% of cases. Remarkably, 85% of IFDs occurred in the first 6 months after starting ibrutinib and 61% occurred in the first 3 months. This trend suggests the possibility that the risk of IFDs, such as IA, decreases with longer exposure to ibrutinib. In the majority of cases, other factors such as corticosteroids, neutropenia, or combined immunochemotherapy that potentially contributed to decreased anti-fungal responses were present. The phenomenon of the high risk of cerebral localization of IA during ibrutinib therapy was also shown in a phase Ib study of ibrutinib treatment of primary central nervous system (CNS) lymphoma; that study reported a 39% incidence of IA in patients who concurrently received corticosteroids in the absence of neutropenia ^30^. To assess the role of BTK in the risk of *Aspergillus fumigatus* infections, the authors evaluated the outcome of IA by comparing the effect of an experimental *A. fumigatus* infection via pharyngeal aspiration in 26 BTK knockout and 20 wild-type mice. Overall, 27% of BTK knockout mice and no wild-type mice died after *A. fumigatus* infection, and more severe lung tissue damage and fungal burden were assessed by histology, indicating a contribution of BTK to the innate immune control of *Aspergillus* infection. These findings suggest that BTK inhibition by ibrutinib impairs innate fungal immune surveillance and that the unusually frequent involvement of the CNS by the fungal infection may be related to CNS macrophage inhibition by the kinase inhibitor. It should be noted that co-administration of dexamethasone or chemotherapy (or both) may exacerbate this effect.

For idelalisib, all studies reported a high rate (from 20 to 40%) of severe infectious complications, particularly during the first months of treatment, and pneumonia was the most frequent disease ^31– 35^. However, in these trials on patients with relapsed or refractory lymphoproliferative disease who received idelalisib, in contrast to trials on ibrutinib, IA was never reported. To the best of our knowledge, only anecdotal cases have been published ^36, 37^.

In conclusion, data from the available literature show that IA is a major infectious complication of ibrutinib treatment, particularly in patients with relapsed or refractory hematologic disease who receive corticosteroids. Although no benefit is expected from the universal use of anti-fungal prophylaxis, patients receiving ibrutinib should be closely monitored and IA should be considered when a pulmonary infiltrate or a cerebral lesion is documented ^14^. In any case, drug–drug interactions between azoles and ibrutinib limit widespread primary prophylaxis. Conversely, available data seem to show that idelalisib therapy does not increase the risk of IA regardless of other concurrent risk factors.

## Anti-apoptotic protein Bcl-2 inhibitors

The elevated expression of the anti-apoptotic protein B-cell lymphoma 2 (Bcl-2), encoded by the gene of BCL2, renders CLL cells and cells of other lymphoproliferative diseases resistant to apoptosis, resulting in the accumulation of long-lived clonal lymphocytes that characterize the disease. Venetoclax is a highly selective inhibitor of Bcl-2 and is indicated in the treatment of CLL patients who have received at least one prior therapy. Neutropenia was the most common grade 3 or 4 toxicity reported with venetoclax administration, but it was managed with either dose interruption or reduction, with or without granulocyte colony-stimulating factor, and the rate of febrile neutropenia was low ^38, 39^. The rate of OIs did not appear to be higher with venetoclax compared with control arms. A comprehensive analysis of the safety of 400 mg daily venetoclax monotherapy in 350 patients with CLL used an integrated dataset from three phase I/II studies: OI occurred in 11 patients (3.1%) and only two cases of pulmonary IA were documented ^40^. In conclusion, according to the published studies, IA does not seem to represent a significant complication in patients with CLL during treatment with venetoclax. Studies on the use of venetoclax in the treatment of NHL, multiple myeloma, and ALs are ongoing, and the available literature does not seem to show a significantly increased risk of infections, including IFD, in these populations ^41– 44^.

## Other kinase inhibitors

Other kinase inhibitors (that is, BCR-ABL inhibitors and Janus kinase inhibitors) have been used for many years for the treatment of chronic myeloproliferative disorders with variable associated infectious complications. According to an extensive literature, these drugs do not expose patients to an increased risk of *Aspergillus* infections ^13, 14^.

## Agents targeting lymphoid or myeloid cell surface antigens

Over the last two decades, there has been increasing interest in developing monoclonal antibodies targeting different surface proteins on cells of lymphoid and myeloid lineages for the treatment of leukemia, lymphoma, and multiple myeloma. The safety profile of agents targeting the lymphoid and myeloid (CD19, CD20, CD52 CD22, CD30, CD33, and CD38) surface proteins were recently reviewed, and recommendations for infection prevention were suggested ^15, 16^. Overall, the treatments were not associated with intrinsic increased risk of infection when compared with controls. In general, most adverse events, including infections, were related to the underlying hematologic malignancy phase and previous treatments. Although various OIs (herpetic infections and *P. jirovecii* pneumonia) may occur in patients who receive the above monoclonal antibodies and targeted agents, cases of IA have been only occasionally or never reported.

## Chimeric antigen receptor-modified T cells and antibodies to immune checkpoint molecule immunotherapy

Immune modulation or “immunotherapy” is a promising therapeutic modality for a variety of cancers. Immunotherapy using targeted chimeric antigen receptor-modified T (CAR-T) cells or antibodies to immune checkpoint molecules represents a dramatic advance in cancer treatment and in particular in the treatment of certain hematologic malignancies. These therapies mediate immune-related adverse events which may mimic or increase the risk of several infections ^45^.

CAR-T cells use gene transfer technology (such as lentiviral or retroviral vectors) to re-program autologous or allogeneic T cells to express CARs, thereby re-directing their specificity to target specific tumor antigens ^46^. Once infused, T-cell products can expand and remain detectable for months to years ^47– 49^. Among hematologic malignancies, CD19-targeted CAR-T cells administered after lymphodepletion chemotherapy constitute a novel treatment for patients with refractory or relapsed B-cell malignancies, including acute lymphoblastic leukemia (ALL), CLL, and NHL. Tisagenlecleucel is the first CAR-T therapy approved for use in children and young adults with refractory or in a second or later relapse of B-ALL ^47, 49^. Tisagenlecleucel and axicabtagene ciloleucel have been used in the treatment of refractory large B-cell lymphoma with high rates of durable responses ^50, 51^. Infectious risk after CAR-T cell therapy is related to the CAR-T toxicity but also to prior therapy. Although a first-line strategy is foreseeable, most of the efficacy and safety data of CAR-T cell treatments involve patients with relapsed or refractory disease at high infectious risk regardless of the salvage immune therapy. A common and challenging complication of CAR-T therapies is the cytokine release syndrome (CRS), which is often characterized by high fever, hypoxia, hypotension, acute kidney injury, transaminitis, and multiorgan dysfunction. Most infections following CAR-T therapy occur during neutropenia or following severe CRS or both ^45, 52^. Infections generally occur within the first 10 days after CAR-T infusion and, when associated with CRS, may be severe and poorly responsive to antimicrobial therapy.

Infections occurring between days 0 and 90 in 133 patients with ALL, CLL, and NHL treated with CD19 CAR-T cells in a phase 1/2 study at the Fred Hutchinson Cancer Research Center were reviewed ^53^. There were 43 infections in 30 (23%) of 133 patients within 28 days after CAR–T cell infusion. Between 29 and 90 days after CAR–T cell infusion, 23 infections occurred in 17 (14%) of 119 evaluable patients. Overall, eight IFDs occurred in six patients (3%), all of whom had severe CRS or neurotoxicity requiring specific treatment and five of whom were autologous or allogeneic HSCT recipients. In three cases, a mold infection was documented: one patient developed a severe *A. fumigatus* sinusitis and an acute pulmonary hemorrhage due to invasive *Aspergillus ustus,* and tracheobronchitis was the primary cause of death in another patient with CLL without neutropenia who died 90 days after CAR–T cell infusion. Infections occurring within the first 6 months in 53 adult patients with relapsed or refractory B-ALL treated with CD19-targeted CAR T cells in a phase I clinical trial at Memorial Sloan Kettering Cancer Center were retrospectively reviewed ^52^. A mold-active anti-fungal prophylaxis (micafungin, posaconazole, and voriconazole) was administered in 41 (77%) of 53 patients. Overall, 22 (42%) of 53 patients experienced 26 infections within the first 30 days of CAR–T cell infusion and 10 (31%) of 32 patients who attained complete remission developed an infection later. Most of the infections were of bacterial origin, and a probable pulmonary IA was documented in three patients (5.7%) while under micafungin prophylaxis. A CRS grade of at least 3 was predictive of infection, but no specific analysis was performed for the risk of IA.

Biological evidence has shown the crucial role of the host immune system in the control of cancer. This led to the creation of monoclonal antibodies that target immune checkpoint signaling pathways and enhance T-cell cytotoxic activity, thereby inducing tumor cell lysis. Cell surface “immune checkpoint receptors”, which usually prevent excessive or non-specific activation of T cells such as programmed cell death receptor 1 (PD-1) or PD-1 ligand (PD-1L) and cytotoxic T-lymphocyte-associated protein 4 (CTLA-4), are targeted by immune checkpoint inhibitors (ICIs). Among the ICIs, PD-1/PD-1L and CTLA-4 inhibitors showed promising therapeutic outcomes, and some have been approved for certain cancer treatments and others are under clinical trials ^54^.

Antibodies targeting PD-1 and CTLA-4 have been investigated in lymphoid malignancies with varying levels of activity and a favorable toxicity profile ^55– 57^. To date, anti–PD-1 antibodies such as nivolumab and pembrolizumab have been evaluated in the setting of relapsed or refractory disease with encouraging response rates, particularly in classic Hodgkin lymphoma but also in follicular lymphoma and diffuse large B-cell lymphoma ^55– 57^.

The adverse events of ICI therapies are related to T-cell cytotoxicity with consequent tumor lysis, fever, and cell damage. Upregulated immune function may target normal tissues expressing immune checkpoint receptors with a consequent release of inflammatory cytokines, antibody binding, and complement activation on normal cells. Other manifestations may be systemic or organ-specific and possibly life-threatening inflammatory reactions. Toxicities may manifest weeks to months after initial treatment but are generally unusual after 3 months ^45^. Infections are a possible complication of ICI therapies, but distinguishing between infections and inflammatory-related manifestations is a challenge. Furthermore, ICIs may enhance inflammation because of infection with a manifestation similar to that of immune reconstitution inflammatory syndrome (IRIS), and anti-inflammatory treatments for inflammatory-related adverse events may mask clinical findings of infection. The treatment of inflammatory reaction, which usually includes steroids, may favor the development of *P. jirovecii* pneumonia and herpesvirus infection, and screening for tuberculosis, endemic fungi, and viral hepatitis B and C is required prior to these treatments ^18, 54– 57^. According to the results of the published clinical trials, ICI therapy in patients with hematologic malignancies does not increase the risk of major opportunistic fungal infections, including IA, beyond the impacts of underlying malignancy and chemotherapy. However, the well-known difficulties in the diagnosis make the data on the real incidence of IA probably unreliable. A peculiar biological effect of checkpoint inhibitors is represented by the possible increase of the host immune response against infections, including those caused by fungi. Preclinical evidence and an anecdotal description of anti–PD-1 activity against fungal sepsis in humans have been reported ^58, 59^. Checkpoint inhibitors could attenuate the clinical progression of invasive aspergillosis and synergize with anti-fungal therapy ^60^.

## Conclusions and perspectives

The recent introduction of new targeted, biological, and cellular therapies has dramatically changed the outcome and complications of several hematologic malignancies. Infections continue to represent challenging adverse events of these therapeutic options but with clinical and epidemiological findings significantly different from those observed in AL patients undergoing intensive chemotherapy and HSCT recipients. Overall, IFDs, including IA, represent a variable and uncommon complication in these populations, but given the increasing eligibility criteria of these novel treatments (particularly in patients with relapsed or refractory hematologic disease) and the prolonged periods of therapy, a considerable number of unusual cases of *Aspergillus* infections can be expected in clinical practice. Given the variable and prolonged period of infectious risk, mold-active prophylaxis is not recommended, but it seems crucial to maintain a high level of diagnostic suspicion with the awareness that IA is a possible complication, particularly during certain treatments. In the event of anti-fungal prophylaxis or therapy, the phenomenon of drug–drug interactions should be considered when certain drugs are co-administered with triazoles. These anti-fungals are inhibitors of the cytochrome enzymes (in particular, CYP3A4), which modulate the metabolism of certain kinase inhibitors such as ibrutinib, and venetoclax. Decisions on the co-administration of these molecules and on the modulation of the dosages are challenging issues in clinical practice. A limit of the data in the available literature is represented by the fact that most information on IA in populations with OHM (that is, other than AL an HSCT) derives from clinical trials not designed for infection purposes. Furthermore, a large part of this population is represented by outpatients receiving oral therapy for prolonged periods outside the control of treating physicians; this makes adequate epidemiological surveillance even more complex. As a consequence, it is difficult to define infection control measures tailored to the emerging hematologic populations undergoing new targeted treatment strategies. It is therefore necessary to carry out continuous surveillance programs of the infectious complications in order to detect in real time any change in the epidemiology of IA and to establish appropriate and tailored diagnostic and preventive approaches.
